# Supplementary material for: Frailty among people with multiple sclerosis who are wheelchair users
Source: PLoS One. 2022 Jul 15;17(7):e0271688. doi: 10.1371/journal.pone.0271688 (PMC9286252; doi:10.1371/journal.pone.0271688)
Supplement: Attachment — Submitted filename: Response_to_Reviewer.docx [file pone.0271688.s002.docx]

Dear Reviewer,

We would like to thank you for your time and efforts in evaluating our manuscript. We have reproduced your comment below, followed by a response highlighting what changes have been made in the revised manuscript.

Tobia Zanotto, PhD, Laura Rice, PhD, Jacob J Sosnoff, PhD.

Reviewer #1: Thank you for the opportunity to review this paper. It's written excellently and has robust conclusions aligned with the results and data.

My only feedback is that I find the connection between frailty and wheelchair use difficult to comprehend/ appreciate. If someone is using a wheelchair or scooter full time, isn't their chance of falling quite small- how are these patients falling over so much, is it in transition from the wheelchair/ scooter to another sitting state (e.g., to the sofa or bed?) I wonder if it's worth a mention in the intro somehow?

**Reply:** Thank you for the positive feedback and for this important question. Although it may be counterintuitive to think that fulltime wheelchair users may be at high risk of falls, previous research has suggested that, indeed, between 40% and 75% of individuals with MS who use wheelchairs or scooters as the primary form of mobility experience at least one fall in any six-month period (Matsuda PN, et al. Falls among adults aging with disability. Arch Phys Med Rehabil. 2015 Mar;96(3):464-71; Rice et al. Fall prevalence in people with multiple sclerosis who use wheelchairs and scooters. Medicine (Baltimore). 2017 Sep;96(35):e7860.). Particularly, the study by Rice and colleagues (2017) indicated that the majority of falls experienced by this segment of the MS population occur at home during transfers (e.g., in the bathroom). While the etiology of falls is multifactorial, it is plausible that elevated frailty levels may increase the risk of falls by impinging on the ability to transfer (e.g., through muscle weakness). We have now mentioned this additional information in the revised Introduction, as suggested. Please refer to page 4, lines 79-84 in the revised manuscript.
